# Maximizing lentiviral vector gene transfer in the CNS

**DOI:** 10.1038/s41434-020-0172-6

**Published:** 2020-07-06

**Authors:** Morgane Humbel, Mergim Ramosaj, Virginie Zimmer, Sara Regio, Ludiwine Aeby, Sylvain Moser, Alexia Boizot, Mélanie Sipion, Maria Rey, Nicole Déglon

**Affiliations:** 1 Lausanne University Hospital (CHUV) and University of Lausanne, Department of Clinical Neurosciences (DNC), Laboratory of Neurotherapies and NeuroModulation, Lausanne, Switzerland; 2 Lausanne University Hospital (CHUV) and University of Lausanne, Neuroscience Research Center (CRN), Laboratory of Cellular and Molecular Neurotherapies (LCMN), Lausanne, Switzerland; 3 Present Address: Laboratory of Neurotherapies and Neuromodulation, Neuroscience reserach Center (CRN), Lausanne Univeristy Hospital (CHUV), Avenue de Beaumont, Pavillon 3, Lausanne, Switzerland

**Keywords:** Neuroscience, Molecular biology

## Abstract

Gene transfer is a widely developed technique for studying and treating genetic diseases. However, the development of therapeutic strategies is challenging, due to the cellular and functional complexity of the central nervous system (CNS), its large size and restricted access. We explored two parameters for improving gene transfer efficacy and capacity for the selective targeting of subpopulations of cells with lentiviral vectors (LVs). We first developed a second-generation LV specifically targeting astrocytes for the efficient expression or silencing of genes of interest, and to better study the importance of cell subpopulations in neurological disorders. We then made use of the retrograde transport properties of a chimeric envelope to target brain circuits affected in CNS diseases and achieve a broad distribution. The combination of retrograde transport and specific tropism displayed by this LV provides opportunities for delivering therapeutic genes to specific cell populations and ensuring high levels of transduction in interconnected brain areas following local administration. This new LV and delivery strategy should be of greater therapeutic benefit and opens up new possibilities for the preclinical development of gene therapy for neurodegenerative diseases.

## Introduction

The central nervous system (CNS) is a highly complex organ, and our knowledge of its physiological and pathophysiological functions and mechanisms remains incomplete [1]. A genetic origin has been found for some CNS diseases, including Huntington’s disease (HD), familial forms of amyotrophic lateral sclerosis, Parkinson’s disease (PD), and Alzheimer’s disease [1]. The use of gene replacement or gene silencing strategies to target the corresponding disease genes is a promising therapeutic approach for these conditions [2]. Viral vectors are widely used to ensure the efficient, sustained and safe delivery of the genes of interest to the CNS. Zolgensma® (AAV-smn) has recently been approved for the treatment of infants with spinal muscular atrophy [3]. Gene replacement and RNA interference strategies are currently being developed for adrenoleukodystrophy [4], lysosomal storage disorders [5], HD [6], and PD [7].

Neuronal cells are the principal cells targeted and transduced following the intraparenchymal administration of viral vectors. However, both neuronal and non-neuronal cells contribute to neurodegenerative disorders [8]. Thus, viral vectors targeting neurons alone may be insufficient for the effective treatment of brain disorders. Astrocyte-specific viral vectors have been developed for studies of the contribution of astrocytes to such conditions and the mechanisms involved [9]. These vectors were generated by altering the ability of vectors to bind to and enter host cells and by integrating transcriptional and post-transcriptional regulatory elements into the vector. Another challenge in the development of CNS treatments is the degeneration of specific regions of the brain at early stages, and of much larger areas of the brain at later stages observed in most neurodegenerative disorders. The affected areas form interconnected neural circuits and constitute large functional networks [10]. For instance, HD is characterized by a specific vulnerability of the medium-spiny GABAergic neurons of the striatum, although other structures that receive or send projections to/from the striatum also degenerate during disease progression [11].

Transduction of the specific cell populations affected in these brain circuits should be considered, to maximize therapeutic benefits. The most potent viral vectors for CNS applications are adeno-associated viral vectors (AAV) and lentiviral vectors (LV) [12]. Diverse AAV serotypes and variants with high retrograde and/or anterograde transport properties have been described in the last few years. For example, the AAV2-retro variant displays levels of retrograde transport two orders of magnitude greater than those of commonly used AAV serotypes [13]. For LV, the most widely used envelope is the vesicular stomatitis virus G-glycoprotein (VSV-G). However, VSV-G-pseudotyped LV (LV-VSVG) can only transduce cells at the site of injection. Researchers have made use of neurotropic viruses, which display natural retrograde spread during their life cycle to demonstrate that LV pseudotyping with the rabies virus G-glycoprotein (RV-G) leads to retrograde transport and expression in distal neurons [14]. Several groups have developed chimeric rabies/VSV-G envelopes for the pseudotyping and transduction of neurons in projecting areas [15]. Various strains of rabies virus and configurations of the chimera were used in these studies [16]. Hirano and coworkers developed two RV-G/VSV-G fusion glycoproteins: FuG-B (from the Pasteur virus strain), with high levels of retrograde transport HiRet [17–19], and FuG-E, displaying neuron-specific retrograde gene transfer (NeuRet) [20].

We explored the possibility of using LVs pseudotyped with the chimeric glycoproteins FuG/B2 to target neurons and astrocytes. We also used the neuronal connectome [21] and the retrograde transport properties of LV-FuG/B2 following injection into a highly interconnected area of the brain to increase transduction efficiency. We found that this delivery strategy significantly enhanced gene transfer, resulting in a widespread distribution of the vector in large areas of the brain following intrastriatal injection.

## Materials and methods

### Plasmids and lentiviral vector production

The GfaABC1D promoter was kindly provided by Dr. Michael Brenner through the support of NIH grant NS39055 [22, 23]. The GfaABC1D promoter was EcoRI-digested from the pGFA-ABC1D plasmid [22] and cloned into the gateway entry vector EcoRI-digested pENTR4 (Roche Diagnostics, Switzerland). The (B)3 enhancer (431 bp) (B sequence Genbank NG_008401: position 3436-3559) was obtained from Bsu36i/SmaI-digested pUC-GFa2(B)3-nls-LacZ plasmid and ligated into Bsu36i/SmaI-digested pENTR4-GFaABC1D. The GFaABC1D(B)3 promoter, hereafter called G1B3, was then digested with BamHI, treated with Klenow to generate blunt ends (Sigma Aldrich, Switzerland), and cloned into Spe/blunt-digested SIN-cPPT-gateway-WPRE-miR124T transfer vector [24]. We used LV expressing the DsRednuc, mCherry, or GFP fluorescent reporter genes under the control of the mouse PGK1 or GfaABC1D(B)3 promoters, the woodchuck post-transcriptional regulatory element (WPRE) and the miR124 targeting sequence (miR124T). The following LVs were used: SIN-PGK-DsRednuc-WPRE; SIN-PGK-mCherry-WPRE, SIN-PGK-GFP-WPRE; SIN-GfaABC1D-GFP-WPRE-miR124T, SIN-G1B3-GFP-WPRE-miR124T, SIN-G1B3-DsRednuc-WPRE-miR124T, SIN-cPPT-PGK-mCherry-WPRE [24–26]. These LVs were pseudotyped with the VSV-G and FuG/B2 envelopes [18] and produced in HEK-293T cells with a four-plasmid system, as previously described [27].

The Vivapure LentiSELECT 500 kit was used for the small-scale purification of lentiviral particles, as previously described [28] (Thermo Fisher Scientific, Reinach, Switzerland). The Sartobind® ion exchange membrane adsorber technology used in LentiSELECT efficiently and rapidly captures and recovers large viral particles (3000 nm pores). The particle content of viral batches was determined by p24 antigen ELISA (RETROtek, Gentaur, Kampenhout, Belgium). The stocks were stored in 1% BSA in PBS at −80 °C until use.

### Primary cultures and transduction of cortical astrocytes

For astrocyte cultures, P1 C57BL/6 mouse pups (Charles River, Ecully, France) were killed by decapitation. Brain cortical tissues were cut into small pieces and mechanically dissociated by repeated aspiration in a fire-polished Pasteur pipette. The dissociated astrocytes were centrifuged at 500 × *g* for 2 min, and the pellet was resuspended in 1 ml of 10% fetal bovine serum (FBS) in DMEM. The cells collected from three pups were used to seed a single T75 flask containing DMEM (Sigma-Aldrich Chemie GmbH, Buchs, Switzerland), 10% FBS (Life Technologies, Zug, Switzerland), 1% antibiotic-antimycotic solution (Sigma-Aldrich Chemie GmbH, Buchs, Switzerland) and 3.7 g/L sodium bicarbonate, pH 7.2. The medium was replaced with fresh culture medium three days after seeding, and then every 5–7 days thereafter. At the first medium change, the flasks were strongly shaken three times to remove microglia, and washed with PBS to remove dead cells. After 2 weeks in culture, the astrocytes were released by trypsin treatment and used to seed a T125 flask at a density of 100,000 cells/ml. Once 90% confluence was reached, the astrocytes were replated in a 24-well plate coated with 15 mg/L poly-L-ornithine (Sigma-Aldrich Chemie GmbH, Buchs, Switzerland), at a density of 100,000 cells/ml. Astrocyte cultures were infected on day 8 with 100 ng SIN-cPPT-G1B3-GFP-WHV-miR124T or 100 ng SIN-GfaABC1D-GFP-WHV-miR124T, and maintained in culture for 1 week. As a negative control, we used noninfected cells.

### Immunofluorescence staining of primary cultures of cortical astrocytes

Eight days post-infection, the medium was removed and the astrocytes were washed twice with cold PBS and incubated for 20 min at 4 °C with 4% PFA. The coverslips were washed with PBS to remove the residual PFA and blocked by incubation for 1 h in 1x PBS supplemented with 10% normal goat serum (NGS, Interchim, Montluçon, France) and 0.03% Triton X-100 (Fluka, Sigma-Aldrich, Buchs, Switzerland). A rabbit polyclonal anti-GFAP primary antibody (RRID: AB_10013382, 1/800, Dako Schweiz AG, Basel, Switzerland) was diluted in 1x PBS −10% NGS −0.03% Triton X-100 and incubated overnight with the cells at 4 °C. The cells were then washed three times, for five minutes each, with 1x PBS, and incubated for 1 h at room temperature (RT) with a goat anti-rabbit IgG AlexaFluor-594 secondary antibody (1/1000, Invitrogen, Life Technologies, Zug, Switzerland) diluted in 1x PBS −10% NGS –0.03% Triton X-100. Cells were then washed 3 times, for 5 min each, in 1x PBS, and mounted in Vectashield HardSet mounting medium (Vector Labs, H-1500). Images were acquired with a x10 objective on a Zeiss AxioVision epifluorescence microscope (Zeiss, Carl Zeiss Microscopy GmbH, Göttingen, Germany). Quantification of DAPI/GFAP demonstrated that the culture contains >85% astrocytes.

### Measurement of mean fluorescence in primary cortical astrocyte cultures

The coverslip with the fixed cortical astrocytic primary cultures were used to measure the mean fluorescence intensity per cell. Images were acquired using the 10x objective of the Zeiss Axio Imager Z1 upright microscope Zeiss, (Car Zeiss Microscopy GmbH, Göttingen, Germany) setting the same exposure time for all the acquisition sessions (500 ms). The GFP-positive cells were manually delimited and the mean gray level of each one was automatically calculated using the ImageJ software. Graphs representing the distribution of MFI/cell were obtained with GraphPad software (GraphPad Prism version 8.00 for Windows, GraphPad Software, La Jolla California USA, www.graphpad.com). Data are expressed as mean ± SD. G1B3-GFP: *n* = 7; GfaABC1D-GFP: *n* = 3.

### Culture and transduction of HEK-293T cells

HEK-293T cells (CRL11268, ATCC/LGC standards, Wesel, Germany) were cultured in DMEM supplemented with 10% FBS, 100 U/ml penicillin, and 100 µg/ml streptomycin, at 37 °C under an atmosphere of 5% CO_2_ in air. On the day before infection, HEK-293T cells were plated, at a density of 140,000 cells per well, in 12-well plates (Corning, Life Technologies, Zug, Switzerland) and incubated for one day. They were then infected with various amounts of SIN-PGK-GFP-WPRE (5, 10, 25, 50, 100 ng p24). Three days later, GFP expression was assessed in wells containing a coverslip coated with 5 µg/ml poly-D-lysine (Corning, Life Technologies, Zug, Switzerland) in HBSS (Thermo Fisher Scientific, Reinach, Switzerland). The samples were fixed with 4% paraformaldehyde (PAF; Lucerna Chem, Lucerne, Switzerland) in Vectashield with DAPI (Reactolab, Servion, Switzerland).

DNA and RNA were extracted from the other wells with TRIzol® (Thermo Fisher, St-Aubin, France). DNA and RNA concentrations were determined with a Nanodrop spectrophotometer (Thermo Fisher Scientific, Reinach, Switzerland). The entire procedure was performed in DNAse-free conditions, in a clean area free of plasmids and PCR products, to prevent sample contamination.

### Vector copy number (VCN) and RT-qPCR in HEK-293T cells

VCN was determined as previously described [29]. We used qLV-HIVgag primers (forward: TCTCGACGCAGGACTCG; reverse: TACTGACGCTCTCGCACC; probe: Yakima-Yellow-ATCTCTCTCCTTCTAGCCTCZNA4-BHQ1) for the test gene and the housekeeping gene encoding poly (rC)-binding protein 2 (qPCBP2; forward: TTGTGTCTCCAGTCTGCTTG; reverse: AGGTGGTGGTGGTGGTA; probe: FAMCCCTCTCCTGGCTCTAAATGTTGTGT- BHQ1) and created a standard curve (16 pg-2 ng) for SIN-PGK-GFP-WPRE equivalent to a VCN of 0.16–20 in 100 ng gDNA, adjusting the amount of plasmid for the size of the human genome (3.1 Gb per haploid genome) [29]. We used 100 ng of gDNA from HEK-293T cells for qPCR, in duplicate, in 20 µl reaction mixtures. We used the KAPA SYBR FAST qPCR kit (Axon Laboratories, Mont-sur-Lausanne, Switzerland) and 200 nM of each primer, with a standard PCR program of 5 min at 95 °C followed by 40 cycles of 3 s at 95 °C, and 20 s at 60 °C (RotorGene Q, Qiagen, Hombrechtikon, Switzerland). For each sample, we analyzed biological duplicates and technical triplicates. The VCN standard curves were generated and LV insertion sites in samples were quantified by the the ΔΔCT method, with *PCBP2* as the internal calibrator gene.

### RT-qPCR

TRIzol-extracted RNA samples from HEK-293T cells were treated with RQ1 RNase-free DNase (Promega, Dübendorf, Switzerland) to remove any trace of genomic DNA. We then generated cDNA from the RNA with Superscript II (Thermo Fisher, Reinach, Switzerland), according to the manufacturer’s guidelines, and diluted it to a final concentration of 1 ng/µL. We performed real-time quantitative PCR on the cDNA with the Kapa Sybr Fast qPCRMaster mix (Axon Laboratories, Mont-sur-Lausanne, Switzerland), according to the manufacturer’s protocol, with Rotor Gene (Qiagen, Basel, Switzerland) and the following cycle parameters: reaction volume of 20 µl containing 200 nM of both forward and reverse primers recognizing a WPRE sequence (WPRE-Fwd: TGTGGATACGCTGCTTTAATG; WPRE-Rev: CATAAAGAGACAGCAACCAGGA), in a Realplex thermal cycler (Eppendorf, Montesson, France). The values obtained for the GFP mRNA were normalized against the β-actin (ACT-1F: TGAAGGTGACAGCAGTCGGTTG, ACT-2R: GGCTTTTAGGATGGCAAGGGAC) reference gene. Technical duplicates were analyzed.

### Mean fluorescence intensity measurement in HEK-293T cells

The coverslips with the fixed HEK-293T cells were used for measurements of the mean fluorescence intensity per cell. Images were obtained with a digital camera (3CCD Hitachi HV-F202SCL) on a slide scanner microscope (10x objective, Zeiss Axioscan Z1). Integrated optical density was measured with Zen 2 image analysis software (blue edition). GFP labeling was quantified by measuring the density of GFP immunoreactivity on 10 regions of interest (300,000 µm^2^). The nonspecific optical density of staining was subtracted from the optical density to calculate an optical density corresponding specifically to GFP.

### Animals

The animals were housed in a controlled-temperature room with a 12 h day/12 h night cycle. Food and water were available *ad libitum*. Animals were transferred to our animal facility 10 days before surgery to allow them to adapt to the new environment. All experiments were carried out in accordance with the European Community directive (86/609/EEC) for the care and use of laboratory animals and Swiss animal welfare laws, under authorization nos. VD 2888, 3073, and 3270 from the *Service de la Consommation et des Affaires Vétérinaires du Canton de Vaud*, Switzerland.

Transgenic mice carrying a bacterial artificial chromosome encoding the enhanced green fluorescent protein (EGFP) under the control of the D2 dopamine receptor promoter (Drd2-EGFP) were generated by the GENSAT (Gene Expression Nervous System Atlas) project [30]. The GLT1-EGFP transgenic mice expressing GFP in astrocytes were kindly provided by Prof. Jeffrey D. Rothstein [31].

### In vivo experimental design and animals

Concentrated viral stocks were thawed on ice and resuspended by repeated pipetting. The mice were anesthetized with 75 mg/kg ketamine/10 mg/kg xylazine, administered intraperitoneally. The SIN-G1B3-DsRednuc-WPRE viral vector (*n* = 3; 200 ng p24 antigen; 2 µl) was injected stereotaxically into the striatum of nine-week-old GLT1-EGFP mice. We injected pCCL-cPPT-H1-shGFP (*n* = 5 per group; 300 ng p24 antigen; 2 µl) into GLT1-EGFP mice and Drd2-EGFP mice. The SIN-cPPT-PGK-cherry-WPRE (150 ng p24 antigen/site) and SIN-cPPT-PGK-GFP-WPRE (150 ng p24 antigen/site) viral vectors pseudotyped with the FuG/B2 envelope were co-injected into the striatum (bilaterally) of C57Bl6 mice (*n* = 4). Finally, the purified batch of SIN-PGK-GFP-WPRE (2 × 300 ng p24 antigen; 5 µl) pseudotyped with the FuG/B2 envelope was injected unilaterally into the striatum of C57Bl6 mice (*n* = 4).

In all cases, injections were performed with a 34-gauge blunt-tip needle linked to a Hamilton syringe (Coopers Needle Works, Birmingham, England) by a polyethylene catheter, at the following stereotaxic (Kopf model 953, Phymep, Paris, France) coordinates: 0.5 mm rostral to bregma, 2 mm lateral to midline, and 3.5 mm from the skull surface. The final experiment was performed with a unilateral injection into the striatum at the following coordinates: site 1: +1.2 mm rostral to bregma, +2 mm lateral to midline, and −3.2 mm from the skull surface, site 2: −0.26 mm rostral to bregma, +2.8 mm lateral to midline, and −3.2 mm from the skull surface, tooth bar: 1.45. The various LVs were injected at a rate of 0.2 µl/min or 0.5 µl/min for the final experiment, with an automatic injector (Harvard Apparatus, Les Ulis, France), and the needle was left in place for an additional 5 minutes. The skin was closed with 6-0 Prolene sutures (B-Braun, Sempach, Switzerland). In all cases, mice were sacrificed 2–3 weeks post injection. No randomization or blinding was used to allocate the animals to experimental groups and processed them. The exclusion criteria were technical failure during the surgical procedure or post-mortem processing of the samples.

### Tropism of the VSVG-G1B3 vector

We assessed the astrocytic tropism of VSVG-G1B3, by acquiring images with a Zeiss LSM 880 Airyscan inverted confocal microscope (Carl Zeiss Microscopy CmBH, Göttingen, Germany). The same settings were maintained throughout the entire experiment (3 animals, 18 hemispheres, one image per hemisphere): 20X enlargement for *z*-stack imaging (number of stack) of the striatum. The infected astrocytes (DsRed2Nuc^+^, DAPI^+^, and GFP^+^ cells) and non-astrocytic cells (DsRed2Nuc^+^ and DAPI^+^ cells, and GFP^-^ cells) were quantified with Imaris Software. The cells on the images were counted (DAPI > 300, GLT1 between 1919 and 3938, DsRedNuc > 1090). Tropism for astrocytes was then calculated by dividing the number of astrocytes infected with the viral vector (DsRedNuc^+^ and GLT1^+^ cells) by the total number of cells infected with the viral vector (DsRedNuc^+^ cells). This quantification was performed for all samples (*n* = 18 hemispheres) and is expressed as the mean ± SD number of infected astrocytes.

### Quantitative analysis of mCherry/GFP coexpression

For quantitative analyses following the coinjection of mcherry and GFP viruses, images were acquired with a x20 objective on a Zeiss LSM 880 AiryScan inverted confocal microscope (Carl Zeiss Microscopy GmbH, Göttingen, Germany). For the cortex, images of 807 µm by 807 µm were acquired with a *z*-stack of 20 µm. For the striatum, images of 807 µm by 1.58 mm were acquired with a *z*-stack of 20 µm. Acquisition parameters were kept constant for images obtained within the cortex and within the striatum. Images were then converted from.czi to.ims files and imported into IMARIS software 9.3 (Bitplan, RRID: SCR_007370). We acquired two or three images per animal. The total number of transduced cells and the numbers of GFP^+^ and mCherry^+^ cells were counted manually.

### Mean fluorescence intensity (MFI) per cell

We used the 20x objective of the Zeiss AxioImager Z1 upright microscope (Carl Zeiss Microscopy GmbH, Göttingen, Germany) to acquire images of the GFP-positive striatal and cortical mouse brain sections, to cover the entire area and prevent overlap between images. We prevented saturation and maximized the intensity of GFP fluorescence, by adjusting the exposure time for acquisition for each of the two zones while keeping all other parameters the same (striatum exposure time: 45 ms; cortex exposure time: 600 ms). For each acquisition session, saturation was prevented with a pixel fluorescence intensity histogram. The GFP-positive cells were manually delimited and the mean gray level of each cell was automatically calculated with ImageJ software. For a semi-quantitative comparison between the striatum and the cortex, the fluorescence levels for each area were normalized to the same time exposure (300 ms). Graphs representing the distribution of MFI/cell were obtained with GraphPad software (GraphPad Prism version 8.00 for Windows, GraphPad Software, La Jolla California USA, www.graphpad.com). Data are expressed as mean ± SD.

### In vivo VCN quantification

The unfixed perfused brains were extracted and positioned in a cold matrix (separated by 1 mm; Phymep, Paris, France). The brains were then cut into 1 mm sections, which were placed on an ice-cold glass plane (cooled by being placed over dry ice, with ethanol added if it became too cold). For each mouse (*n* = 3; 6 hemispheres), sections corresponding to the injection sites were selected. We did not use staining, and we therefore selected three sections per mouse. A hole-punch sample was then obtained for the left hemisphere (non-injected) and the right hemisphere (injected) of each. These samples were then transferred out of the biosafety level 2 facility, on ice. We added 400 μl TRIzol® (Life Technologies, Zug, Switzerland) to each tube and the tissue was homogenized with a pellet mixer (VWR, Dietikon, Switzerland). The tubes were then frozen at −80 °C until use. RNA and DNA were extracted according to the kit manufacturer’s protocol. RNA was stored at −80 °C and DNA was resuspended by incubation overnight in water at RT and was then stored at 4 °C. VCN was measured as described for the in vitro analysis of HEK-293T cells.

### Histological processing

Two to three weeks after LV injection, the animals were killed by an overdose of sodium pentobarbital and transcardially perfused with PBS followed by 4% paraformaldehyde (PAF) (Fluka, Sigma, Buchs, Switzerland). Brains were removed and post-fixed by incubation in 4% PAF for 24 h and were then cryoprotected by incubation in 20% sucrose (Sigma-Aldrich, Buchs, Switzerland) in 0.1 M PBS for 6 h, before storage in 30% sucrose for 24 h. A cryostat (CM1850, Leica Biosystems, Muttenz, Switzerland) with a freezing stage at −20 °C (SM2400; Leica Microsystems AG, Glattbrugg, Switzerland) was used to cut 25 µm-thick coronal brain sections. Sections from the entire striatum were collected and stored in antifreeze solution (0.2 M sodium phosphate buffer, glycerol 25%, ethylene glycol 30%) in 96-well plates at −20 °C.

Striatal sections from the mice that had received injections were processed for immunohistochemistry for DsRednuc (tdTomato Polyclonal Ab; Sicgen: RRID: AB_2722750). The striatal sections (25 µm) were rinsed at RT in TBS (10 mM Tris pH 7.6, 0.9% NaCl) (3 × 10 min) and blocked by incubation for 1 h in TBST supplemented with 2.5% normal donkey serum. Sections were incubated overnight at 4 °C in a solution containing the primary antibody at a dilution of 1/250. They were washed three times with TBS (10 mM Tris pH 7.6, 0.9% NaCl), for 10 min each, and were then incubated with the Alexa Fluor® 568-conjugated donkey anti-goat secondary antibody diluted 1/1000 (IgG (H+L), highly cross-adsorbed: A-11057; Life Technologies, Zug, Switzerland) for 1 h at RT. Finally, the brain sections were washed three times, for 10 minutes each, in TBS and mounted on slides in Vectashield with DAPI (Reactolab, Servion, Switzerland).

### CLARITY sample preparation and lightsheet imaging

Mice were perfused with 4% PFA and tissues were post-fixed by overnight incubation in 4% PFA. Brains were clarified according to the CLARITY protocol [32], with X-CLARITY, a commercial system for electrophoretic tissue clearing (www.logosbio.com). Brains were immersed in a refractive index matching solution (RIMS) containing Histodenz (Sigma Aldrich, Buchs, Switzerland) for at least 24 h before imaging. After clearing, brains were glued to a holder and immersed in a 10 × 20 × 45 mm quartz cuvette filled with RIMS. The cuvette was then placed in a chamber filled with oil with *n* = 1.45 (Cargille). It was then observed with a dual-sided excitation path, a fiber-coupled multiline laser combiner (405, 488, 561, and 647 nm, Toptica MLE), and a detection path comprising a 42 Olympus MVX-10 zoom macroscope with a 1× objective (Olympus MVPLAPO 1×), a filter wheel (Ludl 96A350), and a scientific CMOS (sCMOS) camera (Hamamatsu Orca Flash 4.0 V3). The excitation paths also included galvo scanners for light-sheet generation and the reduction of shadow artifacts due to light-sheet absorption. The beam waist was also scanned with electrically tunable lenses (Optotune EL-16-40-5D-TC-L) synchronized with the rolling shutter of the sCMOS camera. This axially scanned light-sheet mode (ASLM) resulted in a uniform axial resolution across the field-of-view of 5 μm. Images were acquired with custom-written Python software. *Z*-stacks were acquired at a spacing of 5 or 3 μm with a zoom set to a magnification of ×0.8 or ×2, resulting in an in-plane pixel size of 8.23 μm or 3.26 μm, respectively (2048 × 2048 pixels). The excitation wavelength for GFP was 488 nm and a 530/40 nm emission bandpass filter (BrightLine HC, AHF) was used. A complete description of the mesoSPIM microscope was provided in a previous article [33].

## Results

### Targeting astrocytes with the VSV-G envelope and an astrocytic promoter

**Fig. 1:**
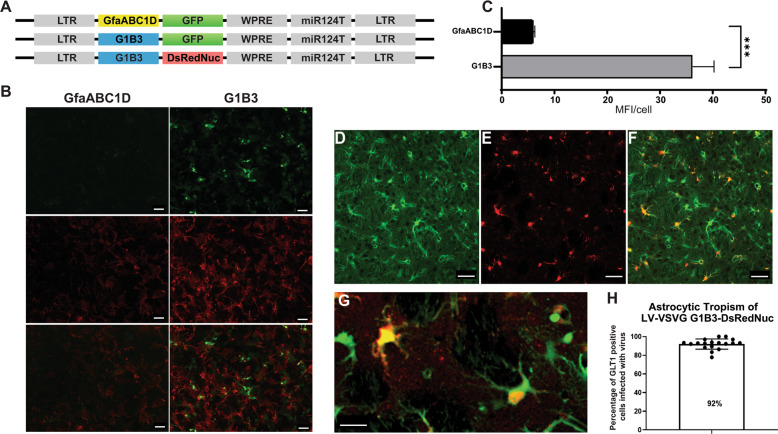
Targeting astrocytes with the VSV-G envelope and an astrocyte promoter. **a** Diagram of the astrocytic LV expressing fluorescent reporter genes under the control of the GfaABC1D and GfaABC1D(B)3 (G1B3 hereafter) promoters and the miR124T targeting sequence. **b** Representative images of cortical astrocyte cultures transduced with GfaABC1D-GFP-miR124T or G1B3-GFP-miR124T LV pseudotyped with the VSV-G envelope (scale bar: 100 μm). Much lower levels of GFP expression were observed with the GfaABC1D-GFP-miR124T than with G1B3-GFP-miR124T. Immunostaining demonstrated that transgene expression was restricted to GFAP-positive astrocytes (red). **c** Transduction of mouse cortical astrocytes using LV encoding a GFP reporter under the control of either a GfaABC1D or a G1B3 promoter demonstrated that the B3 enhancer increased transgene expression. Data are represented as means ± SD and were analyzed with GraphPad Prism. Unpaired *t* test was performed. **d** Images of GLT1-EGFP-positive astrocytes in the striatum of adult mice. **e** Transduced striatal cells expressing DsRedNuc. **f** Merged image of GLT-eGFP^+^ and DsRedNuc^+^ cells (scale bar: 50 μm). (**g**) High-magnification image showing the colocalization of DsRednuc and GFP (scale bar: 20 μm). **h** Analysis of astrocytic tropism with the quantification of DsRedNuc- and GLT1-positive cells expressed as a percentage ± SD.

We and other groups have shown that LVs pseudotyped with the VSV-G envelope and expressing a GFP reporter gene under the control of the PGK promoter have a strong neuronal tropism in rodents [25, 34]. This tropism can be modified toward astrocytic cells through the use of combinations of a heterologous Mokola envelope, an miRNA detargeting strategy [22, 35], and astrocytic promoters [22, 36]. The presence of four copies of the neuron-specific miRNA-124 target sequence (miR124T) in a MOKOLA-pseudotyped LV (MOK/LV) suppresses transgene expression in neurons, but efficiently preserves expression in astrocytes [24]. Similarly, strong astrocytic expression of the transgene was obtained by combining the human glial fibrillary acidic protein (GfaABC1D) [22] or rat glutamine synthetase (GS) promoter with the miR124T sequence [36]. However, these promoters are associated with weak transgene expression in vivo and their activity is barely detectable in primary cultures. Here, we modified the GfaABC1D promoter, integrating three copies of the B enhancer element to improve transgene expression in astrocytes (GfaABC1D(B3), hereafter called G1B3) (Fig. 1a) [22, 23, 26]. The transduction of mouse cortical astrocytes with a LV encoding the GFP reporter gene under the control of the GfaABC1D or G1B3 promoter demonstrated that the B3 enhancer increased transgene expression sixfold (Fig. 1b, c). This result is consistent with the findings of de Leeuw et al., who showed that the integration of the B3 enhancer in the Gfa2(B)3 promoter was 10 times more active than the Gfa2 promoter alone [23].

We then used LV-G1B3-DsRednuc-miR124T to re-evaluate the capacity of the VSV-G envelope for astrocyte transduction. This LV was injected into the striatum of GLT1-EGFP transgenic mice expressing the fluorescent reporter gene in astrocytes (*n* = 3) (Fig. 1d–f). The animals were killed 3 weeks later and immunohistochemical staining for DsRedNuc was performed. Most of the dsRednuc-positive cells colocalized with the astrocytic GFP reporter 91.9 ± 1.3% (Fig. 1d–g). The 8% DsRed2Nuc+DAPI+eGFP− cells are either GLT1-negative astrocytes, or neurons with residual transgene expression as previously demonstrated with other vectors [36]. Altogether, these results demonstrate that the tropism of VSV-G-pseudotyped LV was dictated by the promoters driving transgene expression rather than the VSV-G envelope.

**Fig. 2:**
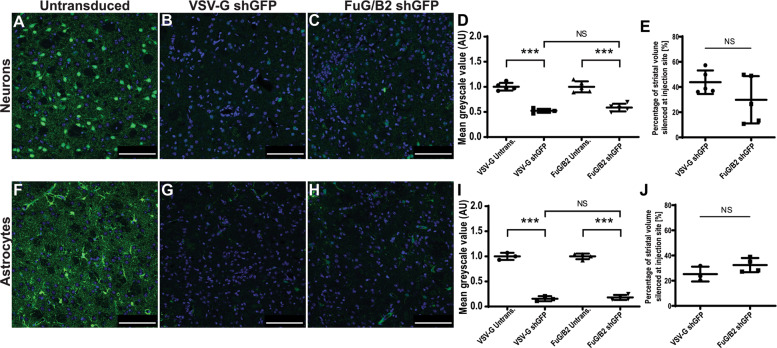
GFP silencing in striatal neurons and astrocytes with LV-VSVG, or LV-FuG/B2. Drd2-EGFP (**a**) or GLT1-EGFP mice (**f**) (*n* = 5; scale bar: 100 μm) mice were injected with pCCL-cPPT-H1-shGFP pseudotyped with the VSV-G (**b**–**g**) or FuG/B2 (**c–h**) envelope into the striatum. The animals were killed 2 weeks later, striatal sections were analyzed and GFP silencing was quantified (**d**, **e**, **i**, **j**). DAPI nuclear counterstain was used on all striatal sections. Data are represented as means ± SD and were analyzed with GraphPad Prism. For **d** and **i**, one-way ANOVA was performed with Tukey post-hoc tests for multiple comparisons. For **e** and **j**, unpaired *t* tests were performed. All data were analyzed with GraphPad. NS non-significant, *P* > 0.05; *P ≤ 0.05; **P ≤ 0.01; ***P ≤ 0.001. The values shown are means ± SD.

We have shown that polymerase III promoters are active in neurons [37]. We further characterized the tropism of LVs pseudotyped with the VSV-G envelope, by assessing the expression of a small hairpin RNA under the control of the H1 promoter in astrocytes. The pCCL-cPPT-H1-shGFP vector was injected into GLT1-EGFP transgenic mice (*n* = 5). As positive control, the vector was injected into Drd2-EGFP transgenic mice expressing GFP in D2 dopamine receptor-positive (Drd2) GABAergic neurons (*n* = 5). Efficient silencing of neuronal GFP expression was observed in the Drd2-EGFP neurons, as previously reported [28, 38] (Fig. 2a, b, d). GFP expression levels were strongly decreased in the astrocytes of GLT1-EGFP mice, as compared with untransduced striatum (Fig. 2f, g, i). These findings demonstrate that LV pseudotyped with the VSV-G envelope efficiently transduces astrocytes and can be used to overexpress or silence genes of interest with polymerase II and polymerase III promoters.

### LV with retrograde transport properties

**Fig. 3:**
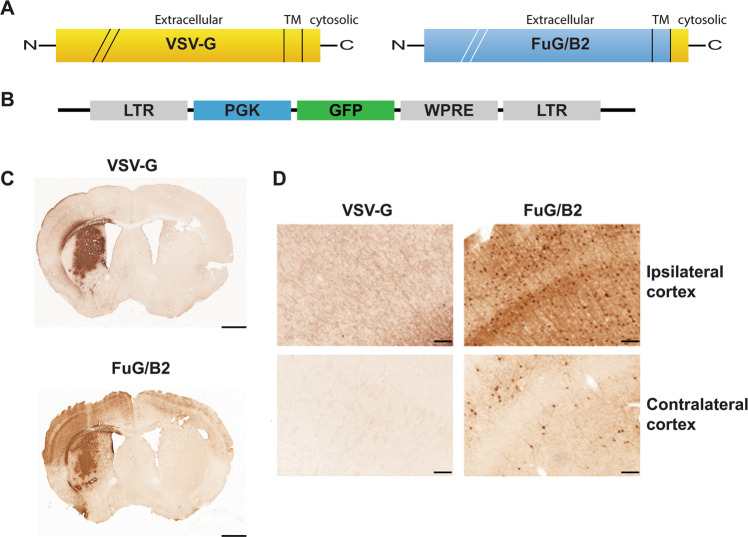
Retrograde transport with the GFP vector. **a** C57BL/6 mice were injecting with either VSV-G (*n* = 3) or FuG/B2 (*n* = 3) envelopes (**b**) expressing green fluorescent reporter gene under the control of the mouse phosphoglycerate kinase 1 (PGK) promoter. **c** GFP-positive cells are present in large areas of the striatum after the injection of VSV-G- and FuG/B2-pseudotyped vectors (scale bar: 1 mm). **d** Consistent with previous reports and the absence of retrograde transport of LV-VSVG, only GFP-positive fibers were detected in the ipsilateral cortex, whereas numerous GFP cells were labeled with LV-FuG/B2 in the ipsilateral cortex, but also in the contralateral cortex (scale bar: 100 µm).

**Fig. 4:**
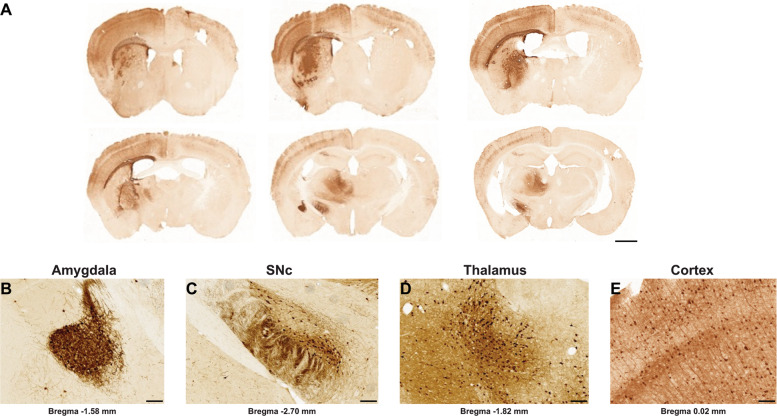
Retrograde transport of SIN-PGK-GFP-WPRE pseudotyped with the FuG/B2 envelope. **a** Coronal sections showing the retrograde transport of LV-FuG/B2 (scale bar: 1 mm). High-magnification images of the (**b**) amygdala (**c**) substantia nigra pars compacta (SNc), (**d**) thalamus and (**e**) cortex (scale bar: 100 µm).

**Fig. 5:**
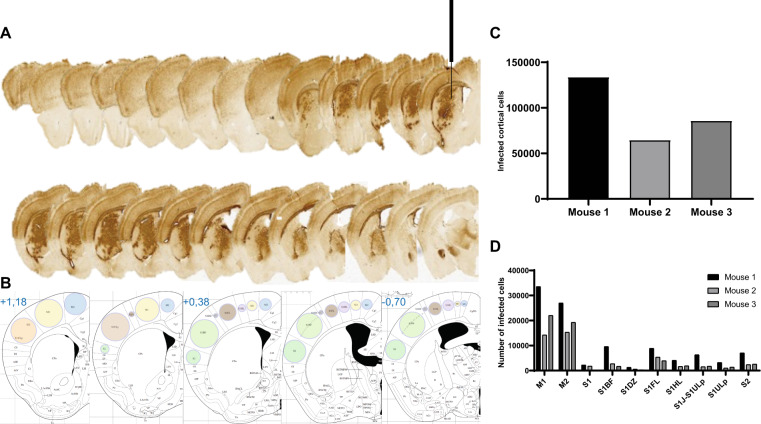
Analysis of retrograde transport in the motor (M1–M2) and somatosensory (S1–S2) cortical areas. **a** Coronal sections showing the retrograde transport of LV-FuG/B2 PGK-GFP reporter from the injection site (see needle) along the entire anterior-posterior axis. **b** Motor and somatosensory cortical cell bodies were quantified from +1.18 to −0.70 mm from the bregma within illustrated subareas described in the Paxinos Atlas. **c** Total infected cell bodies in the motor and somatosensory areas of three mice. **d** Detailed quantification of infected cell bodies within the motor and somatosensory areas of three mice. M1 = primary motor cortex, M2 = secondary motor cortex, S1 = primary somatosensory cortex, S1BF = primary somatosensory cortex, barrel field, S1DZ = primary somatosensory cortex, dysgranular region, S1FL = primary somatosensory cortex, forelimb, S1HL = primary somatosensory cortex, hindlimb, S1J-S1ULp = primary somatosensory cortex, jaw region, and upper lip region, S1ULp = primary somatosensory cortex, upper lip region, S2 = secondary somatosensory cortex.

We used an improved version of the HiRet lentiviral vector, to make use of retrograde transport and neuronal circuitry to reach a large number of CNS regions affected in neurodegenerative disorders. This FuG/B2 envelope is a chimera of the extracellular domain of rabies (Pasteur virus strain) and the transmembrane and cytosolic domain of VSV-G, which efficiently transduces neuronal and glial cells around the injection site and leads to high rates of retrograde transport. GFP fluorescence in the striatum greatly facilitates the quantitative analysis of transduction. We therefore performed a first side-by-side comparison of LV pseudotyped with the VSV-G or FuG/B2 envelope. We injected pCCL-cPPT-H1-shGFP-LV into GLT1-EGFP (*n* = 5) and Drd2-EGFP mice (*n* = 5). The performances of LV-VSVG and LV-FuG/B2 were equivalent in both the neurons and astrocytes of the striatum (Fig. 2). For confirmation of these data and analysis of not only the injection site, but also transduced cells in projecting areas, we performed a second-side-by-side comparison in C57BL/6 mice, with LV-VSVG and LV-FuG/B2 expressing the GFP reporter gene (Fig. 3a, b). GFP-positive cells were visible in the striatum when LV-VSVG was used, confirming previous reports [39]. In the ipsilateral cortex, only GFP-positive fibers were present (Fig. 3b–d). For mice receiving injections of LV-FuG/B2, we identified numerous transduced cells not only in the striatum, but also in the ipsilateral and, to a lesser extent, contralateral cortex (Fig. 3b–d). We investigated the retrograde transport properties of LV-FuG/B2 further, by analyzing regions of the brain projecting to the striatum. Numerous GFP-positive cell bodies were detected in the amygdala (Fig. 4a, b), substantia nigra pars compacta (SNc) (Fig. 4a, c), thalamus (Fig. 4a, d), and cortex (Fig. 4a, e). In the ipsilateral cortex, GFP-positive neurons were observed up to 1.7 mm from the injection site (Fig. 5a). Some variability was observed, probably reflecting the pattern of diffusion of the vector in the striatum in each animal (Fig. 5a, Figure S1). We assessed retrograde transport by quantifying the number of transduced cells in the cortex (17 sections; coordinates +2.68 mm and +0.02 mm from the bregma). We detected 64,637–133,807 GFP-positive neurons (Fig. 5c). The mouse brain atlas was then used as a template to identify the different motor and somatosensory cortical areas and define the somatotopy of retrograde transport (Fig. 5b, Figure S1). The primary motor areas (M1 and M2) were well infected in all animals and the somatosensory areas (S1–S2) were also infected, albeit to a lesser extent (Fig. 5d). These data highlight the potential of the FuG/B2 envelope for efficient gene transfer in large cortical regions, but also the importance of optimizing striatal injection coordinates to maximize retrograde transport.

**Fig. 6:**
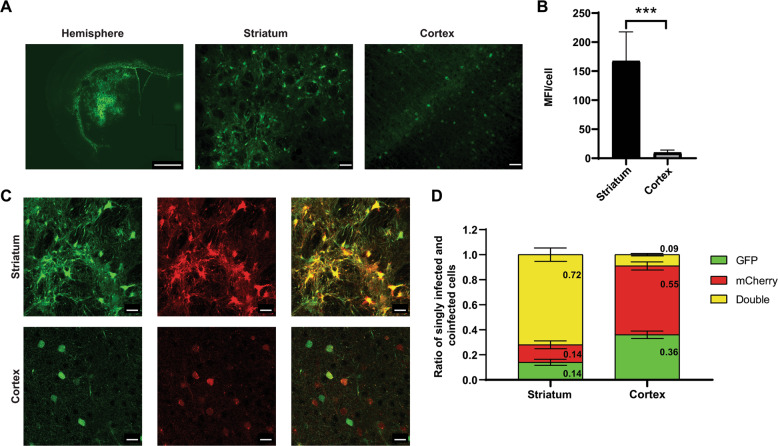
Lower levels of transgene expression in the cortex than at the injection site in the striatum due to the lower VCN in the cortex than in the striatum. **a** Coronal sections showing the retrograde transport of LV-FuG/B2 encoding GFP (scale bar: 1 mm for the hemisphere). High-magnification images of the striatum and cortex (scale bar: 50 µm) (**b**) Quantification of mCherry- and GFP-positive cells in the striatum and cortex of C57/BL6 mice. The data shown are means ± SD. An unpaired *t* test was performed in GraphPad. **c** Images of infected cells in the striatum (upper panels) and cortex (lower panels). Cells are green (GFP^+^), red (mCherry^+^) or yellow (coinfected) (scale bar: 20 µm). **d** Quantification of the cellular portion of single-color and two-color cells indicating that the coinfection was less frequent in the projecting areas than in the striatum.

GFP was expressed more strongly in the striatum than in projecting areas (Fig. 6a), probably due to the presence of a smaller number of vector copies in projecting areas than at the site of injection in the striatum. We tested this hypothesis by co-injecting SIN-PGK-mCherry-WPRE and SIN-PGK-GFP-WPRE (1:1 ratio) pseudotyped with FuG/B2 and analyzing the percentages of mCherry- and GFP-positive cells in the striatum and cortex of C57/BL6 mice (Fig. 6c). We have previously shown that the coinjection of two lentiviruses results in a high proportion of striatal neurons expressing both transgenes [40], and this was confirmed here, with 72 ± 15% striatal cells expressing both fluorescent reporter genes (Fig. 6d). In the cortex, this proportion reached only 9 ± 1%, with the vast majority of cortical cells expressing only the mCherry or GFP protein (Fig. 6d). For further quantification of the levels of retrograde transport and transgene expression levels in the striatum and cortex, we used the GFP reporter gene. The amount of GFP mRNA has been shown to be proportional to the amount of GFP protein [41, 42]. We used the methods developed by Christodoulou et al. [29] to demonstrate correlations between vector copy number (VCN), GFP mRNA level and GFP fluorescence (Figure S2). We infected HEK-293T cells with various amounts of SIN-PGK-GFP-WPRE_VSV-G (5, 10, 25, 50, and 100 ng p24; *n* = 2) and obtained a strong correlation (*R*^2^ = 0.986) between mean fluorescence intensity (protein), GFP mRNA and VCN (DNA) (Figure S2A-D). We used this correlation between MFI and VCN to estimate the striatal/cortical VCN ratio in our experiment. We found that VCN was 16.7 times higher in the striatum than in the cortex (Fig. 6a, b). If we consider the mean VCN in the striatum to be 14 (Figure S2E) (*n* = 3 mice), and assume that not all striatal cells are transduced, then these data suggest a VCN of between 1 and 2 in the cortex, which is consistent with the co-infection data (Fig. 6a). In the last experiment, we optimized the striatal coordinates, based on the mouse cortico-striatal connectome (*n* = 4, Figure 7 = video) [21] and used a purified vector to increase transduction efficiency. For further analysis of the pattern of expression in these animals, and of retrograde transport, in particular, we used an advanced light-sheet microscope to image optically cleared specimens according to the CLARITY protocol (*n* = 2). The native GFP gave strong fluorescence and broad rostro-caudal coverage of the striatum (video). Thus, under optimal conditions, LV_FuG/B2 displays widespread diffusion from the injection site, resulting in high levels of neuronal and astrocytic transgene expression, and is associated with the efficient transduction of projecting neurons.

## Discussion

Clinical trials of gene therapy based on the intraparenchymal administration of viral vectors have reported high rates of neuronal cell transduction [43, 44]. However, the important role of glial cells in neuronal functions and plasticity is increasingly recognized, and advances in brain mapping have revealed the role of neural networks and connectivity in complex motor and cognitive functions [45]. We therefore hypothesize that targeting the glial cells and neuronal circuits affected in neurodegenerative diseases would maximize the therapeutic effect of gene therapy and ensure long-term benefits. We developed a LV capable of efficiently targeting both neurons and astrocytes, with retrograde transport properties. This LV is particularly suitable for the expression of large genes (beyond the cloning capacity of AAV), the simultaneous expression of multiple genes (for lysosomal storage disorders), complex expression systems (large cell-type specific promoters, regulated or self-inactivating systems) and the targeting of dividing CNS precursors.

We first addressed the issue of vector tropism by developing a LV-VSVG, which efficiently transduces astrocytes. We showed that the H1 polymerase III promoter, often used to silence genes of interest, and the G1B3 polymerase II promoter, derived from GFAP, were highly active in astrocytes in vitro and in vivo. The in vivo experiments were conducted in the striatum of adult mice. However, many studies have shown that astrocytes are diverse in terms of both morphology and function [46]. This finding was confirmed by the recent characterization of subpopulations of molecularly different astrocytes [47]. Further studies of the G1B3 promoter will be required to determine its expression profile in different brain regions, astrocyte subclasses, and species. Finally, rapid advances in genomics and massively parallel reporter assays should expand the number of cell type-specific promoter/enhancer sequences and provide new tools for studying glial functions [48].

In gene transfer experiments, the expression profile of the transgene is driven not only by the activity of the promoter, but also by the interaction of viral envelope glycoproteins with host cell surface receptors. In the case of LV, the VSV-G envelope is a trimeric protein (rhabdovirus family) that binds to its cellular receptor and facilitates endocytosis of the virus. Finkelshtein et al. demonstrated that the LDL receptor (LDLR) and other LDLR family members, present in neurons and astrocytes are the major receptors of VSV-G-pseudotyped LV [49]. Envelopes from alphaviruses and other members of the rhabdovirus family have been used to alter the tropism of LVs toward astrocytes. In particular, the glycoproteins derived from the Mokola virus (MOK-G; rhabdovirus) [24, 35], lymphocytic choriomeningitis virus (LCMV; rhabdovirus) [50] and chikungunya virus [51] have been used. However, the high titers of LV-VSVG, the efficient transduction in the CNS of rodent and large-animal models and accumulating preclinical and clinical data have identified LV-VSVG as a candidate of choice for overexpressing or silencing genes of interest in neurons and astrocytes. We show here that LVs pseudotyped with VSV-G efficiently target astrocytes in vitro and in vivo.

The second issue addressed in this study was the development of a lentiviral vector able to reach extensive areas of the brain. This is of particular importance given that neurodegenerative disorders often begin in specific structures of the brain and then propagate to neuroanatomically connected regions. Thus, therapeutic interventions targeting most of the cells involved in these functional and anatomical networks might be more effective for preventing brain damage. We optimized the distribution of the LV in different areas of the brain, by using a chimeric FuG/B2 envelope and optimizing stereotaxic coordinates based on the corticostriatal connectome [21]. According to the projectome data, targeting the dorsolateral and intermediate dorsal and ventrolateral regions of the striatum should favor retrograde transport in cortical regions (primary and secondary motor and sensorimotor areas) of considerable interest for the treatment of HD (Figure S1) [21, 52, 53] and other neurodegenerative diseases affecting locomotion. We found that neurons projecting from the cortex, amygdala, thalamus, and substantia nigra were efficiently targeted by LV_FUG/B2. Mazarakis et al. reported that the retrograde transport of equine infectious anemia virus pseudotyped with the rabies envelope was not restricted to specific neuronal cells [39]. Conversely, not all projection neurons are equally susceptible to retrograde transport for AAV2-retro [13].

The nicotinic acetylcholine receptor, the neuronal cell adhesion molecule, and the low-affinity nerve growth factor receptor (p75NTR) have been proposed as receptors mediating the entry of rabies virus. However, current data suggest that they play a role in the infection process, but probably not in viral entry [54]. Following entry via a clathrin-based dynamin-2-dependant manner [55], the endocytic vesicles containing rabies-G-pseudotyped LV fuse to an early Rab5-positive endosome. Interestingly, for rabies virus, particles that are still enveloped are transported in primary neuronal cultures [56]. However, the precise mechanism involved is still poorly understood. Retrograde transport involves binding to the cytoplasmic dynein motors, which transport cargoes toward the minus end of the microtubules [57]. The sequential activities of Rab5 and Rab7 are required for the coupling of clathrin-dependent endocytosis to fast retrograde axonal transport [58]. Indeed, the conversion of Rab5-positive vesicles into Rab7-positive vesicles may control the generation of axonal retrograde carriers. Rab5 is thought to be responsible for localized axonal movements, whereas Rab7 is responsible for long-range axonal transport [58]. The long-distance axonal transport of AAV9 is also driven by dynein and kinesin-2, and trafficking in a highly motile Rab7-positive compartment [59].

The combined use of the retrograde transport properties of LVs and an optimized surgical and delivery procedure based on projectome/connectome data greatly improve transgene distribution in the CNS and make it possible to target both neurons and astrocytes efficiently. This gene delivery system constitutes a powerful tool for studies of brain connectivity and assessments of the functional contribution of afferents in a circuit. It also opens up new possibilities for the local delivery of LVs to highly interconnected regions of the brain, to achieve a broad distribution and slow the progression of neurodegenerative disorders.

## Supplementary information

Supplemental Figure 1

Supplemental Figure 2

Supplemental material

Video_striatal injection
