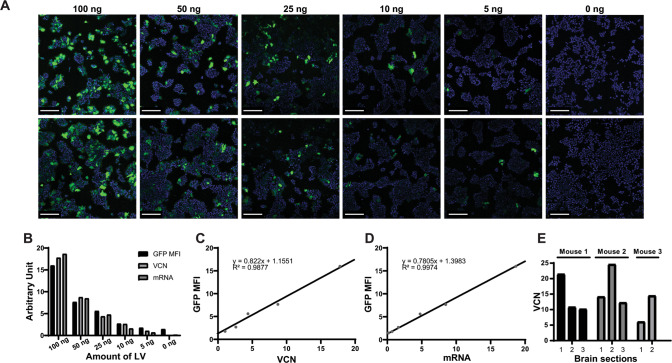# Correction: Maximizing lentiviral vector gene transfer in the CNS

**DOI:** 10.1038/s41434-022-00334-5

**Published:** 2022-04-12

**Authors:** Morgane Humbel, Mergim Ramosaj, Virginie Zimmer, Sara Regio, Ludiwine Aeby, Sylvain Moser, Alexia Boizot, Mélanie Sipion, Maria Rey, Nicole Déglon

**Affiliations:** 1 Lausanne University Hospital (CHUV) and University of Lausanne, Department of Clinical Neurosciences (DNC), Laboratory of Neurotherapies and NeuroModulation, Lausanne, Switzerland; 2 Lausanne University Hospital (CHUV) and University of Lausanne, Neuroscience Research Center (CRN), Laboratory of Cellular and Molecular Neurotherapies (LCMN), Lausanne, Switzerland; 3 Present Address: Laboratory of Neurotherapies and Neuromodulation, Neuroscience reserach Center (CRN), Lausanne Univeristy Hospital (CHUV), Avenue de Beaumont, Pavillon 3, Lausanne, Switzerland

**Keywords:** Neuroscience, Molecular biology

Correction to: *Gene Therapy* 10.1038/s41434-020-0172-6, published online 06 July 2020

In this article, Supplementary Fig. 2 had the wrong information in C and D panels. The correct figure should have appeared as shown below.