# Supplementary figures and images for: Maximizing lentiviral vector gene transfer in the CNS
Source: Gene Ther. 2020 Jul 6;28(1-2):75–88. doi: 10.1038/s41434-020-0172-6 (PMC7902268; doi:10.1038/s41434-020-0172-6)

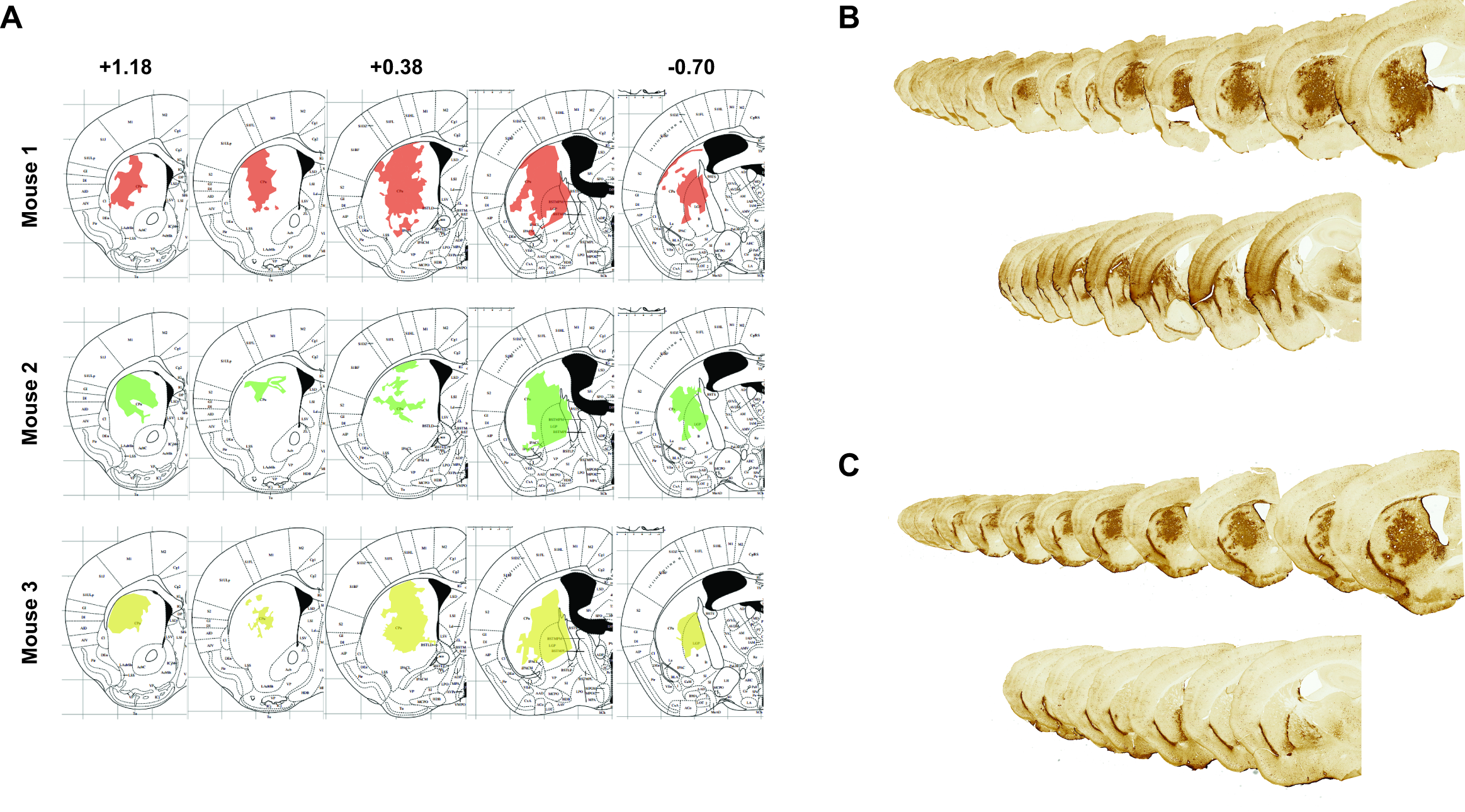

Supplement: Supplementary file 1 — Supplemental Figure 1 [file 41434_2020_172_MOESM1_ESM.tif]

**A**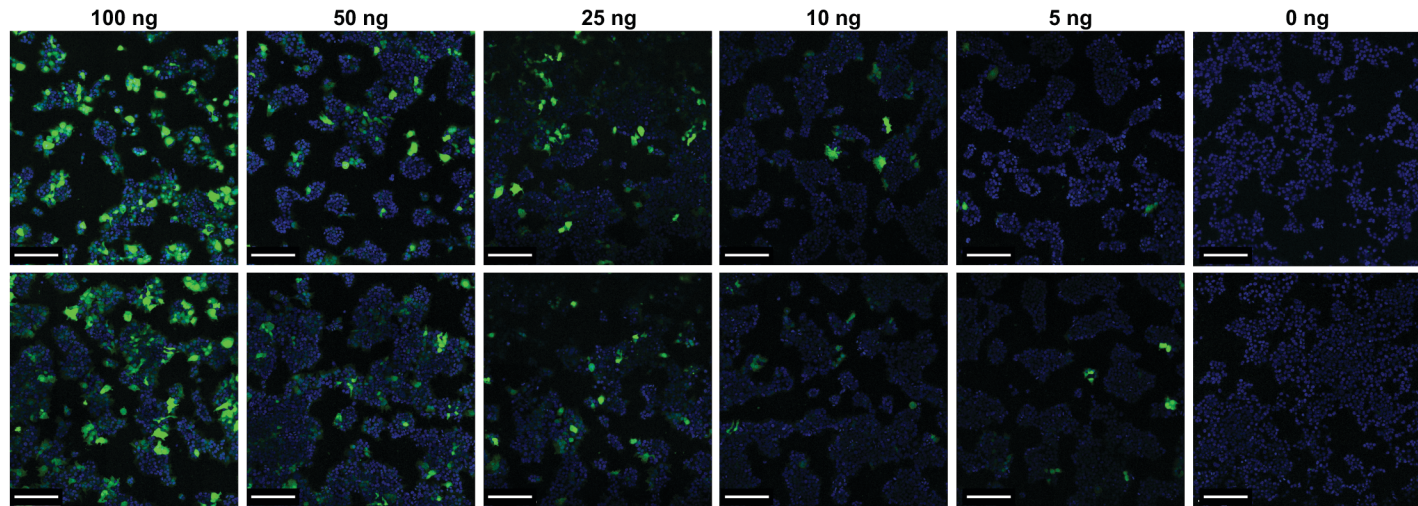**B**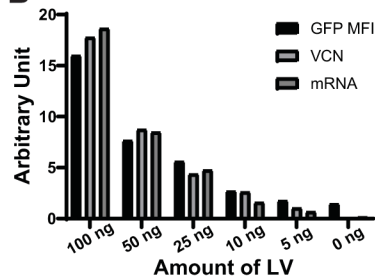**C**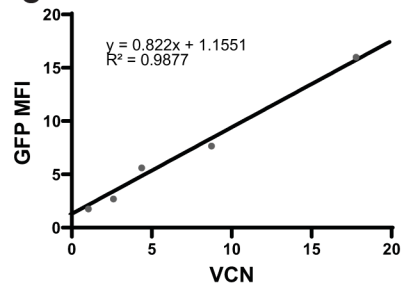**D**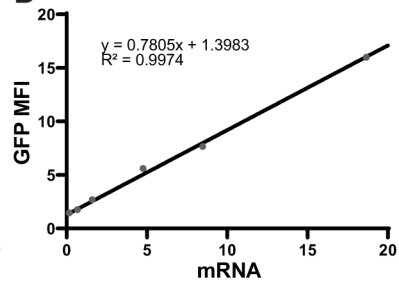**E**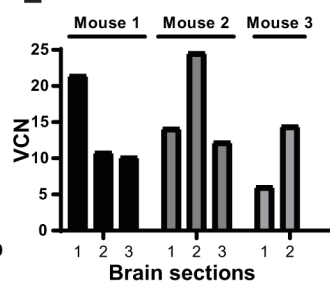

Supplement: Supplementary file 2 — Supplemental Figure 2 [file 41434_2020_172_MOESM2_ESM.pdf]
