# Supplementary material for: Maximizing lentiviral vector gene transfer in the CNS
Source: Gene Ther. 2020 Jul 6;28(1-2):75–88. doi: 10.1038/s41434-020-0172-6 (PMC7902268; doi:10.1038/s41434-020-0172-6)
Supplement: Supplementary file 3 — Supplemental material [file 41434_2020_172_MOESM3_ESM.docx]

Supplementary information

Figure Legends

**Figure S1:**

(**A**) The striatal diffusion of LV-FuG/B2 us illustrated here, from +1.18 to -0.70mm from bregma, according to the Paxinos Atlas. (**B-C**) Coronal sections showing the retrograde transport of LV-FuG/B2 encoding the GFP reporter from the injection site along the entire length of the anterior-posterior axis in two other mice.

**Figure S2:**

(A) HEK-293T cells infected with different amounts of VSV-G encoding GFP: 5, 10, 25, 50 and 100 ng p24 (scale bar: 200 µm). (**B**) GFP MFI, VCN and mRNA were quantified. (**C**) GFP MFI was strongly correlated with VCN (**D**) and mRNA levels. VCN was quantified on brain sections from three mice (*n*=2-3 sections per animal).
